# Developing children’s palliative care in Africa through beacon centres: lessons learnt

**DOI:** 10.1186/1472-684X-12-8

**Published:** 2013-02-18

**Authors:** Julia D Downing, Joan Marston, Casey Selwyn, Laura Ross-Gakava

**Affiliations:** 1 Makerere University, Kampala, Uganda; 2 International Children’s Palliative Care Network, PO Box 7072, Kampala, Uganda; 3 Beacon Centre Coordinator, Cluster Box 3050, 3610, Asagay, South Africa; 4 Research Intern, The Diana, Princess of Wales Memorial Fund, County Hall, Westminster Bridge Road, SE1 7PB, London, England; 5 Palliative Care Initiative, The Diana, Princess of Wales Memorial Fund, County Hall, Westminster Bridge Road, SE1 7PB, London, England

**Keywords:** Palliative care, Children, Africa, Service delivery, Education, Mentors

## Abstract

Much progress has been made in the provision of palliative care across sub-Saharan Africa, however much still remains to be done, particularly in the area of children’s palliative care (CPC). The Beacon Centres programme was set up in 2009, aimed at improving access to CPC in South Africa, Uganda and Tanzania through more and better-trained health professionals and CPC clinical services of a high standard. Having identified sites in each country to develop into CPC Beacon Centres, Navigators were identified who would be the ‘champions’ for CPC in those sites and lead a programme of training, mentorship and support. Five navigators (2 in Uganda and Tanzania and 1 in South Africa) were trained between September and December 2009. Following this they undertook CPC needs assessments at the 3 centres and set up and delivered a six-month CPC training programme, providing mentorship and support to students to enable them to integrate CPC into their workplaces. To date, 188 participants have commenced the six-month course, with 80 having completed it. CPC has been integrated into the activities of the centres and a CPC virtual resource centre set up in South Africa. The achievements from the Beacon project have been great and the work of the navigators immense, but as in all projects it has not been without its challenges. Lessons learnt include issues around: the focus of the project; the length and nature of the training; assessment; accreditation; the choice of navigators; mentoring; administrative support; co-ordination; the choice of project sites; and the integration of CPC into services. The need for CPC is not going to go away and it is therefore important that models of scaling-up are found that are not only practical, feasible, affordable and sustainable, but that focus on the outcome of improved CPC for all those who need it. It is hoped that the lessons shared from the Beacon Project will help in developing and implementing such models.

## Introduction

Palliative care provision within sub-Saharan Africa (SSA) remains for many, a relatively new concept. The Cape Town Declaration in 2002 stated that palliative care is a right of every man, woman and child with a life-limiting disease and that in order to provide good quality palliative care all members of the health care team need training [1]. Much progress has been made since 2002 [2-4] in the provision of palliative care across the region, however much still remains to be done, particularly in the area of children’s palliative care [2,5]. The humane and effective care of children with life-limiting and life threatening disease within SSA is an important public health challenge [6] as nearly 50% of child deaths occurring in developing countries, occur in Africa [7]. Thus there is an urgent need to develop and expand children’s palliative care services in the region.

This paper will discuss the Beacon Centres programme, a programme to develop children’s palliative care services in South Africa, Uganda and Tanzania, through the development of Beacon sites to provide training and clinical experience in children’s palliative care (CPC). The background, implementation and achievements of the project will be discussed along with the lessons learnt that will help guide similar projects in the future.

### Children’s palliative care in Africa

Palliative care for children represents a special, albeit closely related field to adult palliative care. The World Health Organization’s definition of palliative care appropriate for children and their families states that *“it is the active total care of the child’s body, mind and spirit, and also involves giving support to the family. It begins when illness is diagnosed, and continues regardless of whether or not a child receives treatment directed at the disease*” [8].

Whilst there have been many developments in palliative care in Africa over the past few years, developments for palliative care for children continue to lag behind despite the overwhelming need [2,6] and this situation is reflected worldwide [9]. A recent study to map children’s palliative care provision [9] found that of the 53 African countries, 81% had no known children’s palliative care activity, compared to 43% which had no known palliative care activity at all, either for adults or children [4].

Challenges impacting on the development of CPC within Africa include: restricted access to paediatric medical formulations; a lack of understanding of disease processes in children; limited access to affordable and accessible treatment such as chemotherapy or anti-retroviral therapy; limited access to opioids; myths concerning children’s perception of pain; and poverty, forcing parents to make decisions on who to spend their meagre resources on [10]. Providing children’s palliative care is a challenge for many health professionals, and despite the need, they do not feel confident in providing care [5], in particular in terms of communicating with the children and their families [11], and yet there are few local teachers and minimal resources with which to conduct training. Alongside the lack of skilled health professionals, and demonstration sites for care, a review of CPC in SSA [6] found that there is also a lack of research evidence for CPC in Africa related to service development, activity, outcomes and cost of care.

The Beacon Centres programme, funded through The Diana, Princess of Wales Memorial Fund (The Fund), arose out of this need to build capacity, not only for providing CPC, but also to train and mentor health professionals in the field. The goal of the Palliative Care Initiative (PCI) of The Fund is to ensure the integration of palliative care into health systems resulting in palliative care being available to a greater number of people in SSA. They recognised that CPC has been under-developed in SSA and developed a portfolio of work in the area including supporting training in South Africa, funding a paediatric palliative care manager to promote CPC in South Africa, funding the review of CPC services in SSA [6] and the development of a CPC Toolkit [12] and a textbook for Africa [13].

### The beacon centres programme

**Figure 1:**
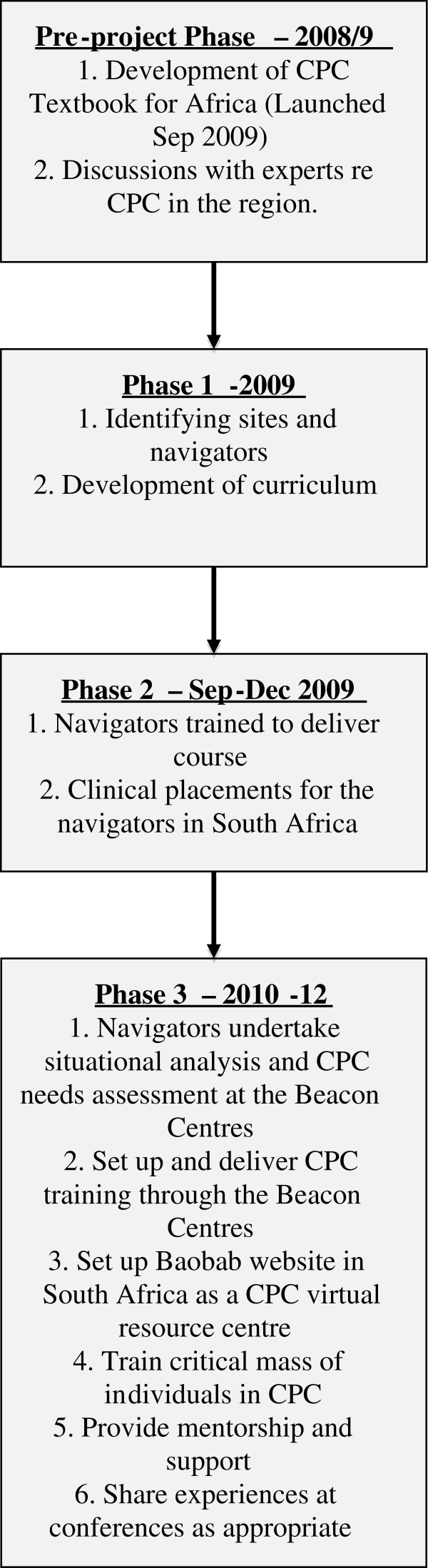
Elements of the Beacon Centres Programme.

The Beacon Centres Programme was set up in 2009 with the objective of improving access to CPC in South Africa, Uganda and Tanzania through increasing trained health professionals to deliver CPC clinical services of a high standard. The focus was to train ‘navigators’ or trainers from different sites to provide ongoing training at the Centres, including clinical placements and mentorship and supervision. Prior to the training each site would undertake a situational analysis and CPC needs assessment. Training materials would be developed by a core group of experts, led by a consultant and the CPC training would run over six months including, two weeks classroom-based training, two-weeks clinical placement and then five months of work-based learning and assessments [14]. Alongside the training a web-based virtual resource would be developed for professionals working in children’s palliative care, particularly focused on South Africa. Following training, participants were encouraged to integrate CPC into their services, and the training was linked to an advocacy strategy to integrate palliative care for children into national policies and undergraduate training [15,16] (Figure 1).

A group of experts from around the region agreed to co-ordinate the project, develop the curriculum and provide training and support. Five potential sites for the centres were identified and three chosen – The Hospice Palliative Care Association of South Africa (HPCA), Mildmay Uganda (Mildmay) and the Pastoral Activities and Services for People with AIDS in Dar-es-Salaam Archdiocese (PASADA) in Tanzania. Criteria for selection included caring for an existing population of children with life-limiting illnesses; support and enthusiasm amongst the staff and capacity to build a team devoted to CPC; access to medications for CPC; willingness to work with external mentors and the ability to offer clinical placements and work towards accreditation of the training programmes.

Two different models were used in the project. Firstly, in South Africa, where there were already CPC services in place, a cluster model (i.e. several beacon centres in different regions, supporting CPC throughout the country) was used based on the St Nicholas Bana Pele Network model in the Free State, supported by the development of the baobab website (http://www.baobabppc.org.za). Secondly, where there were limited or no children’s palliative care services one existing organisation working with children developed as a beacon centre in each country i.e. Mildmay in Uganda (where Hospice Africa Uganda also had a limited children’s palliative care programme) and PASADA in Tanzania. Both models were included as it is important to not only develop services where none exist, but also to support ongoing expansion of services where they do exist and enable national co-ordination of service development and training.

Two navigators were identified at both Mildmay and PASADA, one with a medical and one with a psycho-social background, and one navigator with a medical background in South Africa. Mentorship and supervision for the navigators was carried out by two CPC experts, one from South Africa, who mentored those from PASADA, and one based in England who had previously worked in Uganda who mentored those at Mildmay.

The expectations for the project were high, with a target of 300 people trained in South Africa, and 150 people in both Uganda and Tanzania. It was also anticipated that new CPC services would start up out of the training, however numbers were not specified.

### Achievements and lessons learnt

This programme was ambitious and the first of its kind. Whilst there have been many challenges it was a positive experience and much has been achieved, along with lessons learnt that can help shape future programmes. It became clear as the programme developed, that for sustainability of CPC services, it was important to secure the buy-in of the entire organisation, including administrative and clinical staff. This proved more important for sustainability than reaching the target numbers. The six-months course was found not to be appropriate for everyone, with some people in the organisations needing a shorter programme.

To date, 227 participants have enrolled in the six-month course, with 119 (52%) having completed the course, and 50 (22%) still completing it. 58 participants (26%) have dropped out of the course (having completed the 1-week course), with the majority of these being in South Africa (Table 1). All of the projects have trained health professionals from within and beyond their organisations (who were nominated for training by their managers), and elements of the training are now used in other countries such as Malawi, Kenya and India. The number of drop-outs was high initially in South Africa due to some logistical disorganisation, a lack of follow-up and support and the fact that not everyone needed to complete the six-months training. These issues are being rectified.

**Table 1:** Number of people trained to date through the project

	**1-week course**	**Started 6 month course**	**Completed 6 month course**	**Dropped-out of 6 month course**	**Ongoing students**
**South Africa – HPCA**	214	86	32	46	8
**Uganda – Mildmay**	69	70	44	8	18
**Tanzania – PASADA**	N/A	71	43	4	24
**Total**	283	227	119	58	50

Feedback from participants attending the training has been positive and initial results from an evaluation of the six-months training course [17], suggest that the course objectives and many of the participants’ expectations were met. They report that the course has provided them with the practical palliative care skills essential for providing CPC. Clinicians feel that they have more confidence in providing CPC, and in particular they have the confidence to break bad news to children and how to speak to and involve families in their child’s care. One doctor reported that ‘*before doing the course, I did not feel very confident in dealing with non-medical issues. If someone died, rather than spending time with their family, I just used to go into my office and write the death certificate.’* He now has the confidence both in breaking bad news, and communicating with children’s families [18].

The development of new CPC services takes time but in all three countries there is evidence of new services being set up, as trainees have returned to their places of work and started to integrate CPC into their existing services. During the period of the programme there has been great expansion on the provision of CPC services in South Africa. At the beginning of 2009 there were 19 programmes providing CPC across the country and by June 2011 there were 64 programmes. This was enabled thanks to a combination of the numbers trained and the availability of funding from PEPFAR and The Fund for service development and training. Three services have been identified to develop into Beacon Centres.

Advocacy for CPC continues in each country and the navigators have worked alongside their national associations to advocate for CPC at the national and regional levels. They have also been able to share their experiences through national and international conferences such as the 1^st^ Paediatric Palliative Care Symposium in Lusaka, Zambia in November 2011.

Lessons learnt through the implementation of the programme include:

1. ***Clarification of the focus of the project*** – Within the programme the focus was originally on the training of the navigators and subsequent implementation of the six-months training programme. Whilst this was important, the focus needed to be more on what needs to be done in order to integrate CPC into a small number of targeted organisations, rather than on the training itself.

2. ***The length of the training*** – The in-depth six-month programme is only relevant to some staff and not everyone needs to complete the full six-months. However, in order for individuals to be able to integrate CPC into their services, other personnel in their organisations need to understand and be committed to CPC e.g. other health professionals, managerial staff etc. Therefore it is important to have a variety of training and sensitisation programmes running alongside each other. Thus managers can be sensitised on CPC, some health professionals can attend an introductory course on CPC, and then a smaller number of those committed to developing CPC services will undertake the more extensive six-month programme. There is therefore need for a broader training strategy when developing CPC services.

3. ***The nature of the training*** – Placements and mentorship for students are vital and need to be well planned to enable students to implement what they have learnt [17,18]. There are also differing views on how clinical/ medical the course should be, and this links in to the multi-disciplinary nature of the course. Whilst this is important, the challenges of multi-disciplinary education should not be under-estimated.

4. ***Assessment *–**The final exams for the first group of students relied heavily on multiple-choice questions, which turned out to be a disadvantage to non-English speakers. This has been changed and the focus shifted to more essay based questions along with clinical assessment through ‘Objective Structured Clinical Exams’ (OSCEs) [19].

5. ***Accreditation –***The process of getting accreditation for courses takes longer than was anticipated, yet is important for the ongoing sustainability of the Beacon Centres. Work is ongoing to get the programme accredited in Uganda and Tanzania and the initial two-week course has been awarded Continuing Professional Development points in South Africa, linked to the Free State University.

6. ***Choice of Navigators –*** The navigators are key to the success of a project such as this. One of the key challenges within the African context is the hierarchy that exists between different professions e.g. doctors and nurses. It is important to be aware of the context in which the navigators are working and to adapt for the system as appropriate. It is also helpful if the navigators have prior experience of training to minimise the time needed to set up and run programmes.

7. ***Mentoring*** – The navigators’ mentorship needs vary according to their existing experience, with some needing more input than others. Those with limited support and experience, particularly in relation to training, required more mentorship. An important part of the navigators’ role was also to mentor students once they have completed the six-month programme to help them to integrate CPC into their services – however as the number of students grew this was not sustainable and so there are plans to provide peer-to-peer mentorship by previous students.

8. ***Administrative support****–* Initially the centres were not provided with any administrative support for the project and this was a major issue for them all. This was rectified in early 2011 and a small grant given across the three sites to strengthen the administrative support.

9. ***Co-ordination –*** The co-ordination of the project was a bigger role than had been anticipated. Investment in a full-time project manager would have helped, with clear roles and responsibilities outlined from the start and would have provided more opportunities to travel to the centres for mentorship and ongoing evaluation. There was also a need to facilitate monthly teleconferences, which became a core component of the programme. An annual co-ordination meeting was also held in order to review progress and for forward planning.

10. ***Integration of CPC into services –*** In order to integrate CPC into services there needs to be people in the organisation with not only a sound knowledge of CPC but also knowledge of organisational and service development. Whilst the aim is to build a strong cadre of CPC ‘champions’, it is vital that these individuals have the support of their organisations for the integration of CPC into service delivery. In the long term improved CPC will only take place if the employing institutions themselves are prepared to change their policies and practices.

## Conclusion

There is an ongoing need to develop capacity for CPC within Africa. This programme has demonstrated one model of building capacity and supporting the integration of CPC into existing programmes. Key to the development of a training strategy is the development of programmes that are suitable for the range of individuals needed to support the integration of CPC e.g. managers, health professionals, specific CPC champions etc. Ongoing mentorship and supervision is vital with placements that enable students to implement what they have learnt. Integration of CPC into existing services will involve not only CPC skills, but co-ordination, and a knowledge of organisational and service development.

It is important that models of scaling up CPC are found that are practical, feasible, affordable and sustainable. The Beacon programme is a bold and ambitious programme that has achieved much and the lessons learnt will be useful for organisations interested in developing CPC services.

## Competing interests

The authors declare that they have no competing interests.

## Authors’ contributions

JD drafted and finalised the manuscript. JM and LR-G were involved in the conception and design of the project and ongoing co-ordination and implementation of the Beacon Project. CS reviewed the lessons learnt. All authors critically revised, read and approved the final manuscript.

## License for publication

The corresponding author has the right to grant on behalf of all authors and does grant on behalf of all authors, an exclusive licence (or non exclusive for government employees) on a worldwide basis to the BMC Publishing Group Ltd to permit this article (if accepted) to be published in the relevant BMC journal.

## Pre-publication history

The pre-publication history for this paper can be accessed here:

http://www.biomedcentral.com/1472-684X/12/8/prepub

## References

[B1] Sebuyira LM Mwangi-Powell F Pereira J Spence C The Cape Town palliative care declaration: Home-grown solutions for Sub-Saharan Africa J Palliat Med 2003 6 3 341 343 10.1089/109662103322144646 14509478

[B2] Grant L Downing J Namukwaya E Leng M Murray S Palliative care in Africa since 2005: good progress but much further to go BMJ Support Palliat Care 2011 1 118 122 Published Online First: 6 August 2011doi:10.1136/bmjspcare-2011-000057 10.1136/bmjspcare-2011-000057 24653220

[B3] Powell RA Mwangi-Powell FN Kiyange F Radbruch L Harding R Editorial: Palliative care development in Africa: how can we provide enough quality care? BMJ Support Palliat Care 2011 1 113 114 10.1136/bmjspcare-2011-000101 24653217

[B4] Lynch T Clark D Connor S Mapping levels of palliative care development: a global update 2011 London: WPCA 10.1016/j.jpainsymman.2012.05.011 23017628

[B5] Downing J Marston J Boucher S Children’s palliative care in Africa The Australian Journal of Cancer Nursing 2010 11 2 3 10

[B6] Harding R Sherr L Albertyn R The status of paediatric palliative care in sub-Saharan Africa: An Appraisal 2010 London: The Diana Princess of Wales Memorial Fund

[B7] World Health Organization Shaping the Future 2003 Geneva: WHO

[B8] World Health Organization Palliative care 2002 Available from: http://www.who.int/cancer/palliative/en/ (Accessed 27^th^ January 2012)

[B9] Knapp C Woodworth L Wright M Downing J Drake R Fowler-Kerry S Hain R Marston J Pediatric Palliative Care Provision Around the World: A Systematic Review Pediatr Blood Cancer 2011 56 7 Article first published online: 17 Mar 2011DOI: 10.1002/pbc.23100 10.1002/pbc.23100 21416582

[B10] Mwangi-Powell F Ddungu H Downing J Kiyange K Powell RA Baguma A Ferell BC, Coyle N Palliative Care in Africa The Oxford Textbook of Palliative Nursing 2010 London: Oxford University Press 1319 1329

[B11] Amery J Rose CJ Agupio G A study into the Children’s palliative care educational needs of health professionals in Uganda J Palliat Med 2010 13 2 147 153 10.1089/jpm.2009.0153 19827966

[B12] Marston J Robbertze M Boucher S Children’s Palliative Care Toolkit 2008 South Africa: HPCA

[B13] Amery J Children’s Palliative Care in Africa 2009 London: Oxford University Press

[B14] DPWMF Beacon Project Navigator Training Manual 2010 South Africa: DPWMF

[B15] Downing J Nakawesi J Kiwanuka R Paediatric Palliative Care in Uganda In Knapp C, Madden V and Fowler-Kerry S: Pediatric Palliative Care: Global Perspectives 2012 New York: Springer 41 64

[B16] Marston J Nkosi B Bothma A Knapp C, Madden V, Fowler-Kerry S Paediatric Palliative Care in South Africa Pediatric Palliative Care: Global Perspectives 2012 New York: Springer 27 40

[B17] Agupio G Evaluation of a six months CPC course in South Africa, Uganda and Tanzania 2011 Kampala, Uganda: HAU Report submitted to the DPWMF

[B18] Goyder H Children’s Palliative Care Evaluation Report January 2011 2011 London, UK: DPWMF Report submitted to the DPWMF

[B19] Hodges B Validity and the OSCE Medical Teacher 2003 25 250 254 10.1080/01421590310001002836 12881045

